# L-Theanine Content and Related Gene Expression: Novel Insights into Theanine Biosynthesis and Hydrolysis among Different Tea Plant (*Camellia sinensis* L.) Tissues and Cultivars

**DOI:** 10.3389/fpls.2017.00498

**Published:** 2017-04-07

**Authors:** Zhi-Wei Liu, Zhi-Jun Wu, Hui Li, Yong-Xin Wang, Jing Zhuang

**Affiliations:** Tea Science Research Institute, College of Horticulture, Nanjing Agricultural UniversityNanjing, China

**Keywords:** theanine, biosynthesis, content identification, gene expression profiling, *Camellia sinensis* L.

## Abstract

L-Theanine content has tissues and cultivars specificity in tea plant (*Camellia sinensis* L.), the correlations of theanine metabolic related genes expression profiles with theanine contents were explored in this study. L-theanine contents in the bud and 1st leaf, 2nd leaf, 3rd leaf, old leaf, stem, and lateral root were determined by HPLC from three *C*. *sinensis* cultivars, namely ‘Huangjinya’, ‘Anjibaicha’, and ‘Yingshuang’, respectively. The theanine contents in leaves and root of ‘Huangjinya’ were the highest, followed by ‘Anjibaicha’, and ‘Yingshuang’. The theanine contents in the leaves reduced as the leaf mature gradually, and in stem were the least. Seventeen genes encoding enzymes involved in theanine metabolism were identified from GenBank and our tea transcriptome database, including *CsTS1, CsTS2, CsGS1, CsGS2, CsGOGAT-Fe, CsGOGAT-NAD(P)H, CsGDH1, CsGDH2, CsALT, CsSAMDC, CsADC, CsCuAO, CsPAO, CsNiR, CsNR, CsGGT1*, and *CsGGT3*. The transcript profiles of those seventeen genes in the different tissues of three tea plant cultivars were analyzed comparatively. Among the different cultivars, the transcript levels of most selected genes in ‘Huangjinya’ were significantly higher than that in the ‘Anjibaicha’ and ‘Yingshuang’. Among the different tissues, the transcript levels of *CsTS2, CsGS1*, and *CsGDH2* almost showed positive correlation with the theanine contents, while the other genes showed negative correlation with the theanine contents in most cases. The theanine contents showed correlations with related genes expression levels among cultivars and tissues of tea plant, and were determined by the integrated effect of the metabolic related genes.

## Introduction

L-theanine, or called as γ-glutamyl-L-ethylamide or γ-ethylamino-L-glutamic acid, is a unique non-protein amino acid in tea plant (*Camellia sinensis* (L.) O. Kuntze) (Deng et al., 2008). All theanine that naturally exists in tea plant belongs to L-theanine, D-theanine can only be produced industrially. L-theanine is an important indicator in the quality evaluation of green tea because of the particular umami taste that largely influences the flavor of tea (Yamaguchi and Ninomiya, 2000). The functions of theanine related to food science and human nutrition have been extensively studied since theanine was first discovered in tea plant leaves. Previous studies have shown that theanine can improve memory and learning ability by activating relative central neurotransmitters (Haskell et al., 2008). To a certain extent, theanine can reduce blood-pressure, maintain stability, and promote relaxation and concentration by inhibiting the negative effects of caffeine (Yokogoshi et al., 1995; Kakuda et al., 2000). In addition, theanine performs positive functions in anti-diseases action, including enhancing anti-tumor activity, preventing vascular diseases, and neuroprotection (Rogers et al., 2008; Liu et al., 2009; Zhao and Zhao, 2014). Numerous physiological functions make L-theanine as one of the hot spots in the development and utilization of functional components in tea.

As an important and highly abundant free amino acid in tea plants, L-theanine, almost can be detected in all tissues (Deng et al., 2009). However, L-theanine content in tea plants considerably varies among different cultivars and tissues. For example, the tea plant cultivars with small leaf contain higher amino acid contents than the cultivars with large leaf (Jiang, 2013). The small-leaf tea cultivars are suitable for producing the green tea, and the large-leaf cultivars are used to process the black tea (Wu et al., 2014). In general, the theanine content of the young leaves is the highest, and followed by the old leaves, root, and stem (Selvendran and Selvendran, 1973). Within a year, the theanine content is the highest in spring, and then decreases gradually, after which it rises during autumn to a certain degree. In winter (dormancy period), the theanine in tea plant is accumulated and stored in the root. Until to the March and April of the next year (germination period), theanine shifts to shoots and remains at a relative low level in roots until to August (Takeo, 1979). Moreover, L-theanine content is influenced by natural environmental conditions, such as light intensity and concentration of ammonia (Ashihara, 2015).

The theanine metabolism pathway, including synthesis, transportation, and hydrolysis, has been studied for more than sixty years since theanine was discovered by Sakato (Deng et al., 2010). Numerous pivotal enzymes participate in the metabolism pathway of theanine. The gene expression levels of theanine metabolic enzymes largely influence the content, distribution, and metabolism rules of theanine in tea plants (Shi et al., 2011). In theanine biosynthesis, numerous structural genes encode enzymes that directly catalyze reaction steps that lead to the formation of theanine. The genes encoding TS and GS have been identified and cloned from tea plant, and the sequences of *TS* and *GS* genes present high consistency (Okada et al., 2006; Rana et al., 2008a). The partial coding sequences of GOGAT and GDH have been logged in GenBank. The genes in tea plant encoding to ALT and two tea-specific enzymes, AIDA, and ThYD, are not identified gene sequences (Tsushida and Takeo, 1985; Shi et al., 2011). However, SAMDC and ADC, which are both pivotal enzymes in polyamine metabolism, play similar roles with AIDA.

Ethylamine, one of the hydrolyzates of theanine, is further oxidized to acetaldehyde by AO, which is another precursor of catechin phloroglucinol that participates in catechin biosynthesis (Kito et al., 1968). In addition, NiR, which provides NH_4_^+^ for GS/ GOGAT cycle and α-ketoglutarate reductive amination reaction, performs important functions in theanine synthesis. Interestingly, similar to GS in bacteria, γ-GGT can also catalyze theanine synthesis with glutamine and ethylamine as substrate (Mu et al., 2015).

**FIGURE 1:**
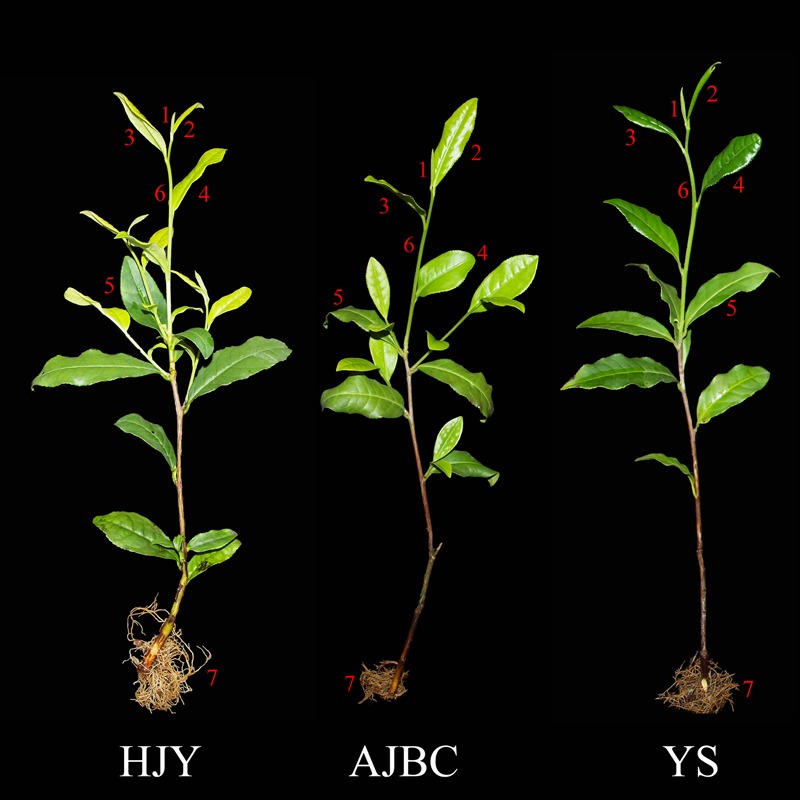
**Three *Camellia sinensis* cultivars ‘Huangjinya’(HJY), ‘Anjibaicha’(AJBC), and ‘Yingshuang’(YS) (1) bud, (2) 1st leaf, (3) 2nd leaf, (4) 3rd leaf, (5) old leaf, (6) stem, (7) root**.

To obtain novel insight into L-theanine metabolism in tea plant, the genes encoding the enzymes involved in the theanine metabolism pathway are identified based on GenBank and our tea plant transcriptome (Wu et al., 2014). The expression patterns of seventeen metabolic-related genes and L-theanine content in the bud and 1st leaf, 2nd leaf, 3rd leaf, old leaf, stem, and root of the three selected tea cultivars were detected and analyzed by RP-HPLC method. The three *C*. *sinensis* cultivars, ‘Huangjinya’, ‘Anjibaicha’, and ‘Yingshuang’, were used in this experiment (**Figure 1**). Based on the obvious difference of geographic and climate characteristics in these three tea production areas, the morphology and physiology of the three tea plants were different. The theanine contents and the ability of adaptation to a single or multiple stresses were different among the three tea plant cultivars. The mainly characteristics of the three tea plant cultivars were listed in **Table 1**. This work is aimed to predict the relationship between structural genes and L-theanine content and the distribution at transcript level, and to provide reference for the further study of regulation mechanisms of theanine metabolism.

**Table 1:** The characteristics description of the three *Camellia sinensis* cultivars, ‘Yingshuang’, ‘Anjibaicha’, and ‘Huangjinya’.

*C. sinensis* cultivar	Breeding method	Variation	Shoot color	Characteristics	Resistance
Yingshuang	Artificial breeding	Non	Green	Lower ratio of polyphenols and amino acids	Strong cold resistance (Zhang et al., 2014)
Anjibaicha	Natural variation	Low temperature- induced albino	White (below 20°C)	High levels of amino acids, low levels of chlorophylls, catechins, and caffeine (Li et al., 2011; Feng et al., 2014)	Sensitive to low temperature (Li et al., 2015a)
Huangjinya	Natural variation	Light-induced albino	Yellow (1.5∼6^∗^10^4^ lux)	High levels of amino acids (Wang et al., 2012)	Sensitive to low temperature and glare

## Materials and Methods

### Plant Material Cultivation and Sampling

Two-year-old cutting seedlings of three *C. sinensis* cultivars (‘Huangjinya’, ‘Anjibaicha’, and ‘Yingshuang’) were selected for this study (**Figure 1**). The tea plants were grown in a matrix composed of peat, vermiculite and perlite (volume ratio = 3:2:1) under the natural environment. On April 2016, the samples of the bud and 1st leaf, 2nd leaf, 3rd leaf, old leaf, new stem (no lignification), and lateral root of three tea cultivars were harvested, immediately frozen in liquid nitrogen, and stored at -80°C.

### Determination of L-Theanine Content

The bud and 1st leaf, 2nd leaf, 3rd, old leaf, new stem, and lateral root of tea plants from three cultivars were dried until constant weight in an oven at 80°C for 24 h. According to the method of GB/T 23193-2008 (China), L-theanine was extracted from different tissues separately, and then theanine content was determined by Reverse-Phase High-Performance Liquid Chromatography (RP-HPLC). The Agilent 1200 Series (Agilent Technologies Co., CA, USA), a Zorbax Eclipse XDB-C18 analytical column (250 mm × 4.6 mm inner diameter, 5 μm nominal particle size), and G1314A UV detector were used for chromatographic separation and detection. The volume ratio of mobile phase A (20 mmol⋅L^-1^ ammonium acetate) and mobile phase B (20 mmol⋅L^-1^ ammonium acetate: methanol: acetonitrile = 1: 2: 2, volume ratio) ratio was modified to 3:2 to better separate the peak in stems and roots.

### RNA Isolation and cDNA Synthesis

RNA was extracted from the buds, leaves, stems, and roots of tea plants according to the instruction manual of the Quick RNA isolation Kit (Huayueyang Biotech Co., Ltd., Beijing, China). RNA concentration and quality were detected by NanoDrop spectrophotometer and agarose gel electrophoresis (12 g⋅L^-1^). One microgram of each RNA sample (ng⋅μL^-1^) was reverse transcribed into the first strand cDNA, after which gDNA was removed by using the PrimeScript^TM^ RT reagent Kit (TaKaRa Biotech Co., Ltd., Dalian, China). Synthesized cDNA was stored at –20°C for quantitative real-time PCR (qRT-PCR) analysis.

### Gene Identification and Primer Design

Nucleotide sequences of genes were searched in the GenBank of NCBI. Genes that are not accessed from NCBI were searched through our transcriptome databases of tea plants (Wu et al., 2014). Genes were further identified by BLAST in NCBI by using the deduced amino acid sequences. Then reads per kilobase per million mapped reads (RPKM) of identified genes in three tea plant cultivars (*C. sinensis* ‘Chawansanhao’, ‘Ruchengmaoyecha’, ‘Anjibaicha’) were searched from transcriptome and were log2 transformed.

Sequences possessing a highly conserved domain and high similarity with other species were selected for qRT-PCR. The primer pairs of identified genes for qRT-PCR were designed by Primer Premier 5. Principles of primer design were as follows: temperature range of 55°C to 65°C, lengths in the range of 18–24 bp, and GC content of 45 to 60 %.

### qRT-PCR Analysis

qRT-PCR was performed using an SYBR Premix *Ex Taq* kit (TaKaRa Biotech Co., Ltd., Dalian, China), iQ^TM^5 software, and iQ^TM^5 qRT-PCR system. The cycling profile is as follows: denaturation at 95°C for 5 min; 40 cycles at 95°C for 5 s, and 60°C for 30 s; and 61 cycles of melt curve analysis at 65°C for 10 s. The 20 μL total volume of the reaction system contained 10 μL of SYBR Premix *Ex Taq*, 7.2 μL of double-distilled H_2_O, 0.4 μL each of primer, and 2 μL of diluted cDNA. Each independent RNA and corresponding cDNA sample was analyzed in triplicate for each tea plant cultivar and each tissue. Each reaction was technically repeated for three times to ensure the accuracy.

### Statistical Analysis

The mean values and standard deviation (SD) of theanine contents and gene mRNA levels were calculated based on three independent biological replicates. The gene expression levels were calculated relative to the *CsTBP* gene by the 2^-ΔΔC^_T_ method (Livak and Schmittgen, 2001). The gene mRNA level of bud and 1st leaf in ‘Yingshuang’ was defined as 1. Significant differences in theanine contents and gene mRNA levels were detected by Duncan’s multiple-range test at the 5 % level with SPSS 17.0 software. The column chart was performed with Origin 6.0 software.

## Results

### Tea Plants of the Three *C. sinensis* Cultivars

Candidate tissues, including the bud, 1st leaf, 2nd leaf, 3rd leaf, old leaf, stem, and lateral root from three *C. sinensis* cultivars, ‘Huangjinya’, ‘Anjibaicha’, and ‘Yingshuang’, were selected to detect of the L-theanine contents, respectively (**Supplementary Figure S1**). The three tea plant cultivars are shown in **Figure 1**, and the shoots of ‘Anjibaicha’ turned into the early re-greening phase from the albescent phase.

### L-Theanine Contents in Different Tissues among the Three *C. sinensis* Cultivars

**FIGURE 2:**
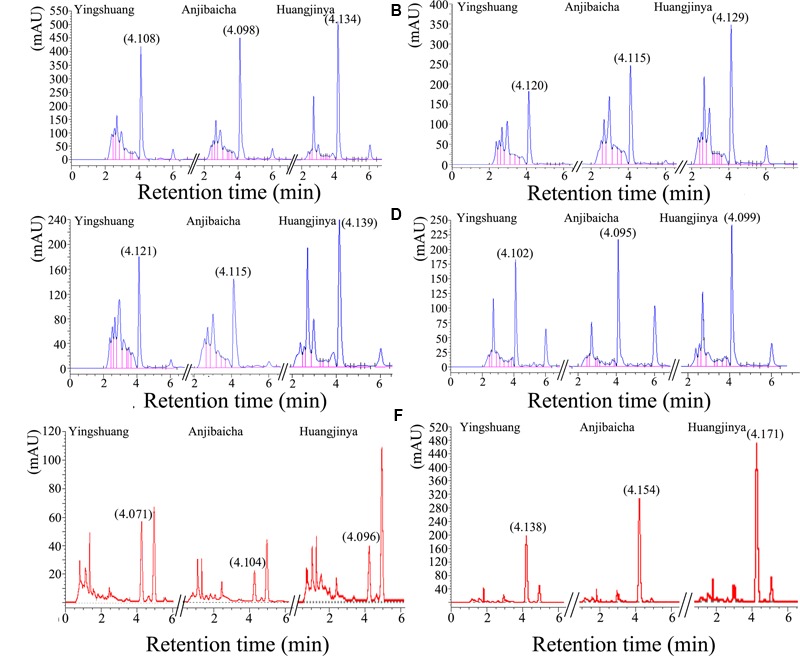
**The high-performance liquid chromatography (HPLC) profiles of different tissues in three *C*. *sinensis* cultivars. (A)** The bud and 1st leaf, **(B)** 2nd leaf, **(C)** 3rd leaf, **(D)** old leaf, **(E)** stem, **(F)** root.

The HPLC profiles of theanine in different tissues among the three *C. sinensis* cultivars were shown in **Figure 2**. The profiles of leaves, stem, and root were different, the retention time were all almost at 4.1 min. In addition, the height of profiles showed positive correlation with the area, except the 3rd leaf of ‘Anjibaicha’. The detailed information of HPLC profiles and profiles of standard samples were listed in **Supplementary Table S1** and **Figure S2**.

**FIGURE 3:**
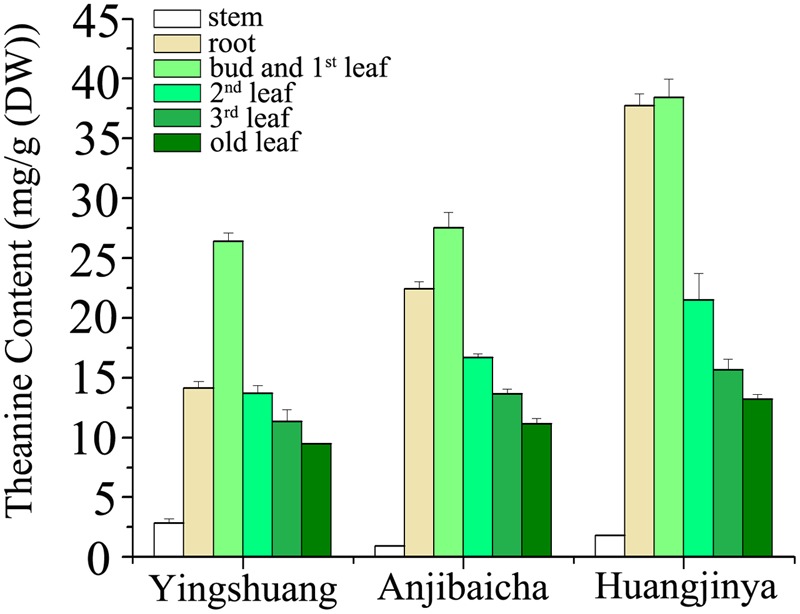
**L-theanine content in various tissues of three *C. sinensis* cultivars.** Values are the means of three independent experiments and are calculated as mg theanine equivalents per 1 g DW (mg/g).

The L-theanine contents show evident difference among the three *C. sinensis* cultivars (**Figure 3**). Among the three tea plant cultivars, ‘Huangjinya’ showed the highest theanine content in the leaves and roots, whereas ‘Yingshuang’ contained the lowest content. The theanine contents of the bud and 1st leaf, 2nd leaf, 3rd leaf, mature leaf, and roots in ‘Huangjinya’ were 1.5-, 1.6-, 1.4-, 1.4-, and 2.6-folds of ‘Yingshuang’, respectively. However, the maximum theanine content in stems was in ‘Yingshuang’, whereas the minimum content was found in ‘Anjibaicha’.

The theanine content of stem was considerably below that of leaves and roots (**Figure 3**). Comparatively from bud to root, the bud and 1st leaf presented the highest theanine amount among the tissues, and the stem showed the lowest. In ‘Huangjinya’ and ‘Anjibaicha’, the theanine content in bud and 1st leaf was approximate to that in root. Theanine content in leaves decreased gradually with the position of leaves moving downward among all three tea cultivars. In general, the theanine distribution among the different parts of the three tea cultivars was similar extremely.

### Gene Encoding the Enzymes Involved in L-Theanine Metabolism in Tea Plants

**FIGURE 4:**
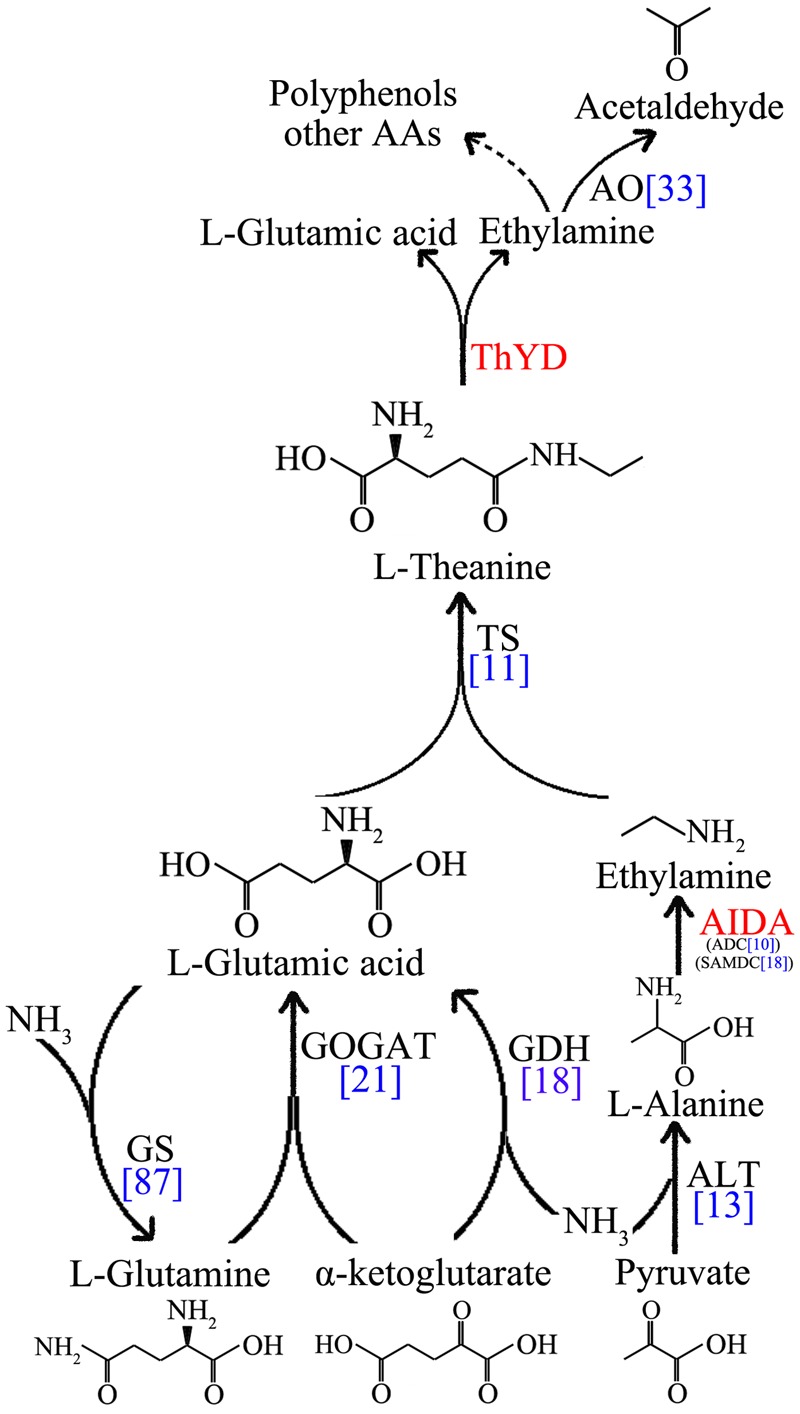
**Schematics of the L-theanine metabolism pathway.** Enzymes that are not identified in tea plants are marked in red. The unigenes number of theanine metabolic enzymes are marked in blue.

Genes encoding the metabolic enzymes involved in catalysis of theanine synthesis and hydrolysis were identified and analyzed in tea plant. *CsTS, CsGS, CsAIDA, CsGOGAT, CsGDH*, and *CsALT* as structural genes encode the enzymes implicated in the synthesis of theanine. *CsThYD* and *CsAO* genes encode enzymes that hydrolyze the synthesized theanine (**Figure 4**).

However, *CsAIDA* and *CsThYD* genes code specific enzymes in tea plant with no orthologs from other species at present (Shi et al., 2011). Thus, the genes were not annotated and identified in our database. Here, the transcripts of *CsSAMDC* and *CsADC* genes, sharing similar domains with *CsAIDA*, were found in the transcriptome (Zhang et al., 2015). In addition, NR and NiR, which oxidize the nitrate to ammonium by collective effect, are also required for theanine synthesis. Ammonium is indispensable to the GS/GOGAT cycle and in the reductive amination of α-ketoglutarate. GGT, which was used to synthesize theanine in bacteria, also exists in tea plants.

In this study, a total of 11, 87, 9, 12, 18, 13, 33, 18, 10, 9, 1, and 3 putative transcripts for *CsTS, CsGS, CsGOGAT-Fe, CsGOGAT-NAD(P)H, CsGDH, CsALT, CsAO, CsSAMDC, CsADC, CsNR, CsNiR*, and *CsGGT* were identified from the transcriptome (**Table 2** and **Supplementary Table S2**). Seventeen genes were identified for further study by BLASTP, including *CsTS1* (GenBank ID: DD410895.1) and *CsTS2* (GenBank ID: DD410896.1) two genes searched from the GenBank, and fifteen genes (*CsGS1, CsGS2, CsGOGAT-Fe, CsGOGAT-NAD(P)H, CsGDH1, CsGDH2, CsALT, CsSAMDC, CsADC, CsCuAO, CsPAO, CsNiR, CsNR, CsGGT1*, and *CsGGT3*) verified from the transcriptome (**Table 3** and **Supplementary File S1**, **Table S3**). The nucleotide and amino acid sequence alignments of those fifteen theanine metabolic related genes among tea plant and other species were listed in **Supplementary Table S4**.

**Table 2:** Numbers of unigenes involved in the theanine biosynthesis pathway.

Enzymes	Number of unigenes
SAMDC	18
ADC	10
ALT	13
GDH	18
AO	33
NR	9
NiR	1
GOGAT-Fe	9
GOGAT-NAD(P)H	12
GGT	3
TS	11
GS	87

**Table 3:** L-theanine metabolic related genes in tea plant.

Gene family	Annotation	GenBank ID/Unigene ID	Sequence length (bp)	Encoded enzyme number
*TS*	*TS1*	DD410895.1	1071	EC 6.3.1.6
	*TS2*	DD410896.1	1071	
*GS*	*GS1*	T4_ BMK.28374	1068	EC 6.3.1.2
	*GS2*	T2_ BMK.52923	1296	
*GOGAT*	*GOGAT-Fe*	T2_ BMK.52873	4881	EC 1.4.1.14
	*GOGAT- NAD(P)H*	T4_ BMK.68558	5583	
*NR*	*NR*	T1_ BMK.80048	2160	EC 1.7.1.1
*NiR*	*NiR*	T4_ BMK.66088	1761	EC 1.7.7.1
*GGT*	*GGT3*	T2_ BMK.38431	1884	EC 2.3.2.2
	*GGT1*	T4_ BMK.61745	1824	
*GDH*	*GDH2*	T2_ BMK.12564	1239	EC 1.4.1.3
	*GDH1*	T1_ BMK.76899	1233	
*ALT*	*ALT*	T4_ BMK.66138	1623	EC 2.6.1.2
*AO*	*CuAO*	T1_ BMK.69752	2355	EC 1.4.3.6
	*PAO*	T1_ BMK.39464	2037	
*SAMDC*	*SAMDC*	T2_ BMK.35350	1077	EC 4.1.1.50
*ADC*	*ADC*	T4_ BMK.63973	2169	EC:4.1.1.19

**FIGURE 5:**
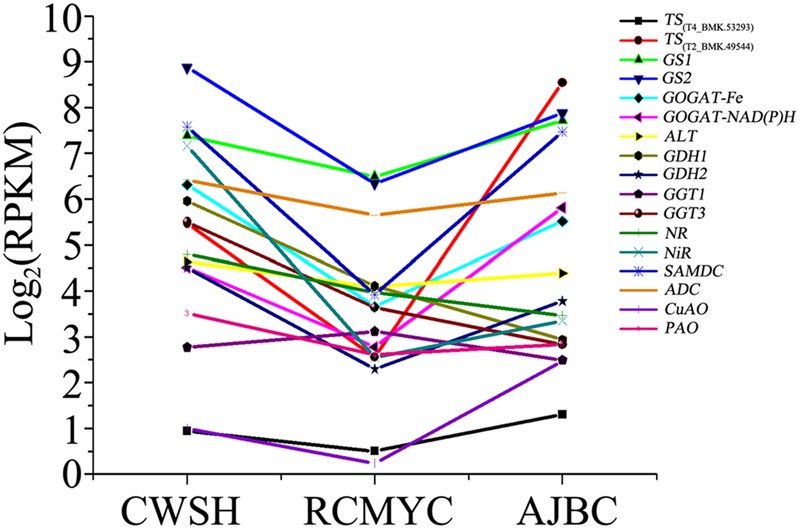
**Expression levels of L-theanine metabolism pathway genes among *C. sinensis* cultivars ‘Chawansanhao’, ‘Ruchengmaoyecha’, and ‘Anjibaicha’**.

Then the unigene expression changes in different tea plant cultivars after quantification using the RPKM values (**Figure 5** and **Supplementary Table S5**). Among the selected 17 unigenes, except the *CsGDH1, CsGGT1, CsGGT3*, and *CsNR*, most of them expresses lower level in the *C. sinensis* ‘Ruchengmaoyecha’ (large-leaf cultivar) than that in the other two cultivars *C. sinensis* ‘Chawansanhao’ and ‘Anjibaicha’ (small-leaf cultivar). It may be deduced that the expression of metabolic genes involved in theanine metabolism were correlated with theanine content to a large extent.

### Expression Profiles of Genes Involved in L-Theanine Metabolism in Different Tissues of Three *C. sinensis* Cultivars

**FIGURE 6:**
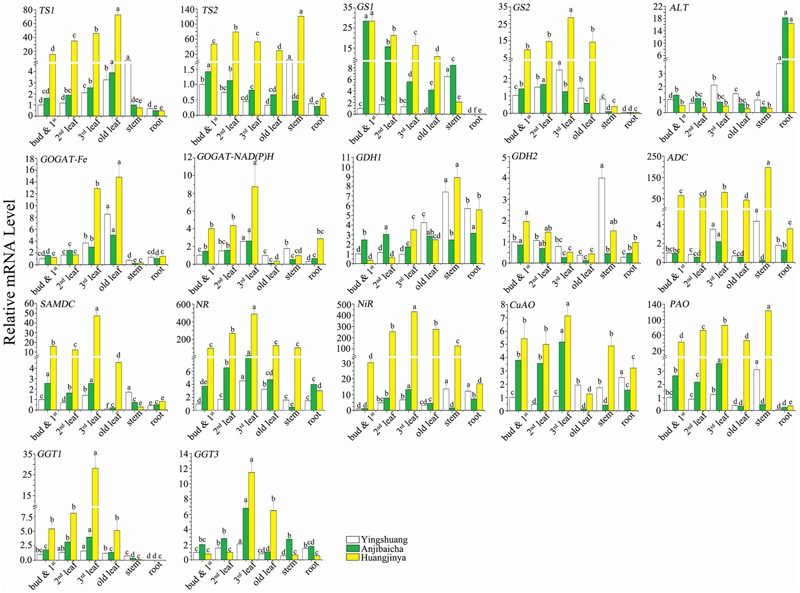
**Expression profiles of L-theanine metabolism related genes among various tissues of *C*. *sinensis* cultivars ‘Huangjinya’, ‘Anjibaicha’, and ‘Yingshuang’.** The gene mRNA level of bud and 1st leaf in ‘Yingshuang’ is defined as 1. The different lowercase letters on the bar graph indicate significant differences at *P* < 0.05.

To further validate the correlation between gene expression level and theanine content, the expression profiles of the genes encoding to the enzymes involved in theanine metabolism were detected in different tissues of three *C. sinensis* cultivars by using qRT-PCR. The expression profiles of the theanine metabolic genes among different tissues and different cultivars are shown in **Figure 6**. Nucleotide sequences of primer pairs used for qRT-PCR that are specific to each theanine metabolic related gene are given in **Table 4**.

**Table 4:** Nucleotide sequences of primers specific to L-theanine metabolic related genes and *CsTBP* gene used for qRT-PCR.

Gene	Forward primer 5′–3′	Reverse primer 5′–3′
*TS1*	AGACCGCCGACATCAACAC	ATGGCTTCCACAGCAGAGT
*TS2*	CCTAAACCTATTGAGGG TGACTG	TCCTGTAAGCCGACGCTCATT
*GS1*	ATCAGTTGTGGATGGCTCG	CACTTCGCATGGACTTGGTAC
*GS2*	GTGGCACCAACGGAGAAGT	CAAGGATGTATCTAGCGCACC
*NiR*	GGACAGGCTGCCCAAATAG	TCACTCCCAATCCTCCCTC
*NR*	TTGATGCTTGGGCTGACA	ACGGACCAGGGATGTGCT
*GGT1*	GATGAATCTTGGTGATCCTGAT	TTCCACCGTCCACCATAGT
*GGT3*	GGAGTCAGCTTCAAGATCACG	CAGTAGGCGTCGAGAAGTCAC
*GDH1*	GAGCTGAAGACATACATGACCA	GCACGAGCAACACGATTAA
*GDH2*	ATGTGGGACGAAGAGAAGGTG	GCAACACGATTCACTCCCAG
*ALT*	CGAGTCCTACGAGTCTTAT TATGC	GGAGGCGTTGACAATAGAATG
*CuAO*	CAGGTTGTTGAGGTGAATGTTA	AATCCAGTGGCGAGCAGA
*PAO*	GTCGGGGTGACGATACCTTAG	CACCACTTAGCGTCGACATTAT
*GOGAT-Fe*	TGCTGGTATGACTGGAGGTT	CAACTGCCAGAATAGCGGTA
*GOGAT- NAD(P)H*	GCAGCGAGGAGATGATTGA	CACCTTCCACATTGGTTGAG
*SAMDC*	TTCCAGCCAAGCGAGTTC	AACCTCCCTCCTTGCCGA
*ADC*	GGGCTTATGAGGAGGCAC	GCAAGAGGGTCCTGGCAT
*CsTBP*	GGCGGATCAAGTGTTGGAA GGGAG	ACGCTTGGGATTGTATTCGG CATTA

#### Expression Profiles in Different Tissues

With the maturity of leaf increases, the transcript abundance of *CsTS1* and *CsGOGAT-Fe* were increased, but that of the *CsTS2, CsGS1*, and *CsGDH2* decreased. The transcript levels of *CsGS2, CsGOGAT-NAD(P)H, CsNR, CsNiR, CsPAO, CsGGT1*, and *CsGGT3* were gradually increased in the bud and 1st leaf, 2nd leaf, and 3rd leaf, but later declined in old leaf. The expression level of *CsADC, CsSAMDC*, and *CsCuAO* in 3rd leaf reached to maximum, then followed in bud and 1st leaf, 2nd leaf, old leaf. The mRNA level of *ALT* presented low and no significant difference in leaves of different parts.

Among the selected genes, most of them showed low transcript abundance in stem and root. The *CsTS1, CsTS2, CsGS1, CsGS2, CsPAO*, and *CsGGT1* showed the lowest transcript abundance in root, but the *CsALT* showed the highest. The transcript levels of *CsGS1, CsGS2, CsGGT1* were close to zero. The stem transcript levels of *CsTS2, CsGDH1, CsADC*, and *CsPAO* reached the highest among the tissues in ‘Yingshuang’ and ‘Huangjinya’. In addition, in stem of ‘Yingshuang’, the expression levels of *CsTS1, CsGDH2, CsSAMDC*, and *CsNiR* were significantly higher than that in other parts.

The genes belonging to same family, such as the *CsGOGAT-Fe* and *CsGOGAT-NAD(P)H, CsGGT1* and *CsGGT3, CsPAO* and *CsCuAO, CsNR*, and *CsNiR*, the transcript levels showed similar trend from bud to root. However, the tendency of expression levels of *CsTS1* and *CsTS2, CsGS1* and *CsGS2, CsGDH1* and *CsGDH2* was almost opposite among different tissues.

#### Expression Profiles in Three Tea Plant Cultivars

Among cultivars, ‘Huangjinya’ showed the highest leaves mRNA levels of most theanine metabolic related genes, moreover the lowest levels were usually observed in ‘Yingshuang’. The gene mRNA levels in ‘Huangjinya’ exhibited tens and even hundreds of times of the lowest levels. However, the leaves expression level of *CsALT*, the bud and 1st leaf and 2nd leaf expression levels of *CsGDH1* and *CsGGT1* in ‘Huangjinya’ were the lowest among three tea cultivars.

Structural genes directly involved in theanine synthesis, *CsTS2, CsGDH1, CsGDH2, CsGS2, CsGOGAT-Fe, CsGOGAT-NAD(P)H*, and *CsADC*, expressed the lowest stem transcript levels in ‘Anjibaicha’. The highest root mRNA levels of *CsTS2, CsGDH2, CsGOGAT-Fe, CsGOGAT-NAD(P)H, CsADC, CsSAMDC*, and *CsNiR* were detected in ‘Huangjinya’. Furthermore, the root mRNA level of *CsALT* in ‘Anjibaicha’ and ‘Huangjinya’ was notably higher than that in ‘Yingshuang’. The expression patterns of the genes from bud to root among different tea cultivars were highly similar.

## Discussion

The L-theanine metabolism pathway involves in the unique accumulation and translocation pathway of nitrogen in tea plant, which belongs to one of perennial economic crops with ammonium resistance (Konishi et al., 1969). At present, a few studies about the enzymes involved in the theanine metabolism pathway at gene level have been reported (Shi et al., 2011; Li et al., 2015b). To better understand the theanine metabolic mechanism at molecular level and provide references for identification and breeding of high theanine tea plant resources, it is essential to explore the correlations between theanine metabolic related genes and contents.

In this study, different tissues of three tea plant cultivars ‘Huangjinya’, ‘Anjibaicha’, and ‘Yingshuang’ were selected, including buds and 1st leaf, 2nd leaf, 3rd leaf, old leaf, stems, and roots. Among different tissues, the stem shows the lowest theanine content, thereby indicating that stems may only act as the transport part of theanine (Oh et al., 2008). In the ‘Huangjinya’ and ‘Anjibaicha’, root theanine content was close to the content of bud and 1st leaf, and higher than previously reported. This finding may be due to variety specificity and weakened transportation of theanine from root to leaves and bud at the re-greening phase of albino tea (Hahlbrock et al., 1971). Among the different cultivars, they presented highly similar distribution tendencies of theanine contents from bud to root. The theanine content in ‘Huangjinya’, ‘Anjibaicha’, and ‘Yingshuang’ decreased by degrees among the tissues except stems, thereby it confirmed that albino tea plants contained higher theanine concentration than non-albino varieties (Li et al., 2011; Feng et al., 2014). Particularly, the high theanine content of ‘Huangjinya’ explained why it is provided with high quality and high popularity.

In qRT-PCR analysis, the high expression levels of most theanine metabolic genes in ‘Huangjinya’ with high theanine concentration. It was basically consistent with the prediction of transcriptome by RPKM values, that is the higher transcripts levels of related genes in small-leaf tea cultivars (‘Chawansanhao’ and ‘Anjibaicha’, higher theanine content) than that in large-leaf cultivar (‘Ruchengmaoyecha’, lower theanine content). In ‘Huangjinya’, the expression levels of *CsNR* and *CsNiR* reached to several hundreds of times higher than the other two cultivars. While the NR and NiR play key role in providing the source of ammonium in theanine synthesis (Yang et al., 2013). In addition, the high expression levels of *CsTS1, CsTS2, CsGS2, CsADC*, and *CsSAMDC* in ‘Huangjinya’ also offered the possibility for synthesis of abundant theanine.

However, the expression patterns of the selected genes among tissues exist difference at different degrees, even though the genes belong to a family. The *CsTS1* and *CsTS2, CsGS1*, and *CsGS2* showed almost opposite correlations with the theanine content in leaves. The transcript levels of *CsTS1* and *CsTS2* in root were lower than in leaves, but in shoot were less than and more than in mature leaves, respectively. Those comparison results among shoot, mature leaves, and root were identical with the results of Deng (Deng et al., 2008). The negative correlation between mRNA levels of *CsGS1* and theanine concentration in tea plant was comparable to that, which was previously reported for rice (*Oryza sativa* L.) and tobacco (*Nicotiana tabacum* L.) transfected *CsGS* gene. In rice and tobacco, the overexpression of transfected *CsGS* gene caused the decrease in free amino acids, including glutamic acid and glutamine (Migge et al., 2000; Cai et al., 2009). The positive correlation between mRNA level of *CsGDH2* and theanine content, possibly because GDH is strongly activated by abundant amide compounds, such as glutamine and theanine, to assist GS/GOGAT (Rana et al., 2008b). In addition, most genes showed lower expression levels in root than that in other tissues. However, the expression level of *CsALT* was up-regulated in root and much higher than that in stem and leaves. The *CsALT* was the key gene in the process from ammonia converted to ethylamine. The ethylamine showed high conversion rate to synthesize theanine in root (Deng et al., 2009). Then the theanine might act as an easily transported nitrogenous compound were transported to other tea plant tissues (Deng et al., 2012). The high expression level of *CsALT* in root was consistent with the high synthesis rate of theanine, and promoted indirectly the transportation of theanine.

In this study, some genes expression level showed positive correlations with theanine contents among different tea plant cultivars, which explained the potential reason of the tea plant cultivar contains high amount of theanine at molecular level. However, some other theanine metabolic related genes exhibited negative correlations among different tissues. Ashihara mentioned that the root of tea plant played important roles in theanine synthesis. The synthetic theanine is preferentially transported and accumulated in the tender tissues (Ashihara, 2015). In addition, the theanine in mature leaves were probably degraded more quickly than in young leaves. Tsushida and Takeo also demonstrated that the degradation products of theanine as substrates were used for the synthesis of other polyphenols and other amino acids (Tsushida and Takeo, 1985).

In addition, we can see the high similarity of expression profiles of *CsGOGAT-Fe* and *CsGOGAT-NAD(P)H, CsADC* and *CsSAMDC, CsNR* and *CsNiR, CsPAO* and *CsCuAO, CsGGT1* and *CsGGT3* among different parts. It may be deduced that they play synergistic roles in regulation of theanine metabolism. Therefore, the theanine metabolism is co-regulated through many related genes, which may apply synergistic or antagonistic effect. The tissue, cultivar, developmental stages, and growth environment also can influence the expression profiles of theanine metabolic related genes, the genes regulation of theanine metabolism became complicated. This study revealed the correlation between theanine content and genes expression to a certain extent, and provide support for the high theanine tea plant germplasm at gene level, as well as molecular theory foundation for quality improvement of tea plants.

## Author Contributions

JZ and ZL initiated and designed the research. ZL and JZ performed the experiments. ZL, ZW, HL, YW, and JZ analyzed the data. JZ contributed reagents, materials, analysis tools. ZL wrote the paper. JZ and ZL revised the paper.

## Conflict of Interest Statement

The authors declare that the research was conducted in the absence of any commercial or financial relationships that could be construed as a potential conflict of interest.

## Supplementary Material

FIGURE S1 **The bud and 1st leaf, 2nd leaf, 3rd leaf, and old leaf from three *C. sinensis* cultivars, ‘Yingshuang’, ‘Anjibaicha’, and ‘Huangjinya’**.

FIGURE S2 **The chromatogram of theanine standard samples. (A)** 0.01 mg/mL; **(B)** 0.02 mg/mL; **(C)** 0.05 mg/mL; **(D)** 0.1 mg/mL; **(E)** 0.2 mg/mL.

FILE S1 **The domains of selected theanine metabolic related genes**.

TABLE S1 **The original data of HPLC profiles**.

TABLE S2 **The annotations of theanine metabolic related genes in tea plant transcriptome**.

TABLE S3 **The annotations of selected theanine metabolic related genes for qRT-PCR**.

TABLE S4 **The nucleotide and amino acid sequence alignments of theanine metabolic related genes among tea plant and other species**.

TABLE S5 **The RPKM and Log_2_(RPKM) values of the selected theanine metabolic related genes**.

## Abbreviations

ADC: Arginine decarboxylase
AIDA: L-alanine decarboxylase
ALT: alanine transaminase
AO: amine oxidase
CuAO: copper methylamine oxidase
DW: dry weight
GDH: glutamate dehydrogenase
γ-GGT: γ-glutamyl transpeptidase
GOGAT: glutamine-2-Oxoglutarate synthetase
GOGAT-Fe: ferredoxin-dependent glutamate synthase
GOGAT-NAD(P)H: NAD(P)H-dependent glutamate synthase
GS: glutamine synthetase
NiR: nitrite reductase
NR: nitrate reductase
PAO: primary amine oxidase
RP-HPLC: Reverse phase high performance liquid chromatography
SAMDC: *S*-adenosylmethionine decarboxylase
ThYD: L-theanine hydrolase
TS: L-theanine synthetase
