# Supplementary figures and images for: L-Theanine Content and Related Gene Expression: Novel Insights into Theanine Biosynthesis and Hydrolysis among Different Tea Plant (Camellia sinensis L.) Tissues and Cultivars
Source: Front Plant Sci. 2017 Apr 7;8:498. doi: 10.3389/fpls.2017.00498 (PMC5383724; doi:10.3389/fpls.2017.00498)

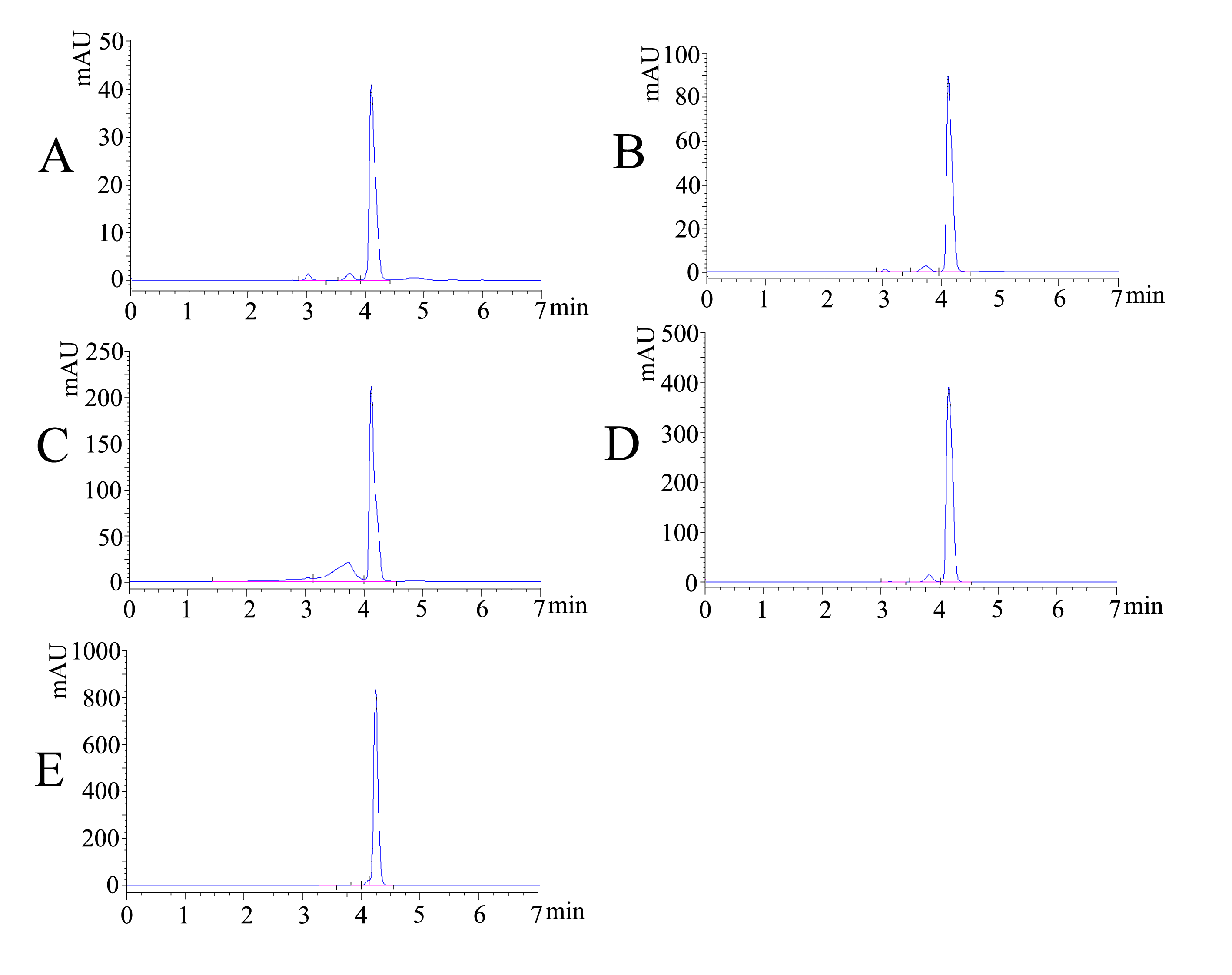

Supplement: FIGURE S1 — The bud and 1st leaf, 2nd leaf, 3rd leaf, and old leaf from three C. sinensis cultivars, ‘Yingshuang’, ‘Anjibaicha’, and ‘Huangjinya’. [file Presentation_1.ZIP › Supplementary Figure 2.tif]

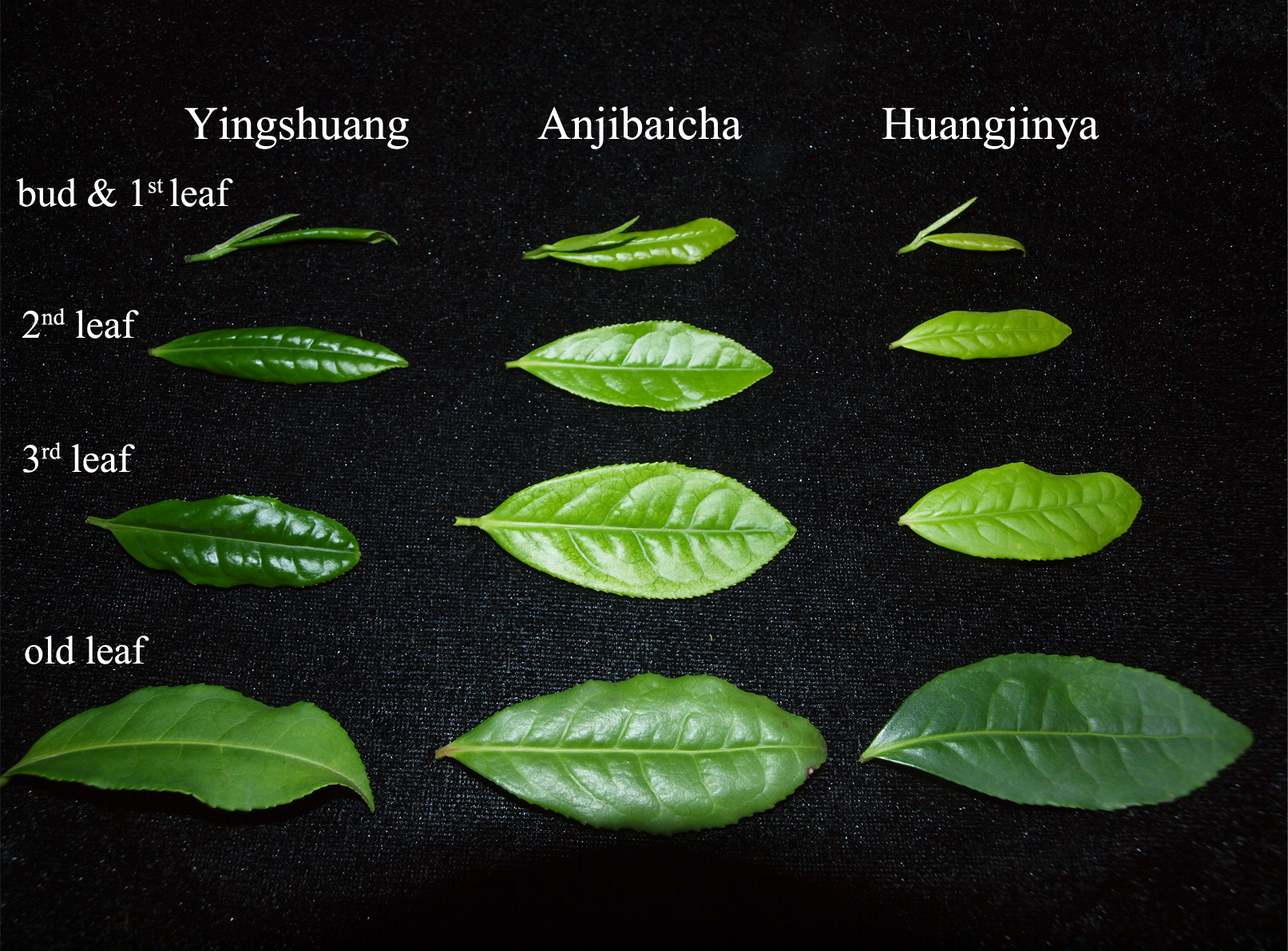

Supplement: FIGURE S1 — The bud and 1st leaf, 2nd leaf, 3rd leaf, and old leaf from three C. sinensis cultivars, ‘Yingshuang’, ‘Anjibaicha’, and ‘Huangjinya’. [file Presentation_1.ZIP › Supplementary Figure 1.tif]
