# Supplementary material for: L-Theanine Content and Related Gene Expression: Novel Insights into Theanine Biosynthesis and Hydrolysis among Different Tea Plant (Camellia sinensis L.) Tissues and Cultivars
Source: Front Plant Sci. 2017 Apr 7;8:498. doi: 10.3389/fpls.2017.00498 (PMC5383724; doi:10.3389/fpls.2017.00498)
Supplement: FIGURE S1 — The bud and 1st leaf, 2nd leaf, 3rd leaf, and old leaf from three C. sinensis cultivars, ‘Yingshuang’, ‘Anjibaicha’, and ‘Huangjinya’. [file Presentation_1.ZIP › Supplementary File 1 The domains of selected theanine metabolic genes.doc]

**Supplementary File 1** The putative conserved domains of the theanine metabolic genes searched from the transcriptome database

***CsGS1*:**

***CsGS2*:**

***CsGDH1*:**

***CsGDH2*:**

***CsGOGAT-Fe*:**

***CsGOGAT-NADH*:**

***CsALT*:**

***CsADC*:**

***CsSAMDC*:**

***CsGGT1*:**

***CsGGT3*:**

***CsCuAO*:**

***CsPAO*:**

***CsNR*:**

***CsNiR*:**
